# The effect of convalescent plasma therapy on the rate of nucleic acid negative conversion in patients with persistent COVID-19 test positivity

**DOI:** 10.3389/fphar.2024.1421516

**Published:** 2024-08-01

**Authors:** Yixuan Wang, Zhe Xu, Xue Xu, Shuwen Yang, Yuanyuan Li, Hanwen Zhang, Yufeng Zhang, Fu-Sheng Wang, Ying Wang, Jingfeng Bi

**Affiliations:** ^1^ School of Pharmaceutical Sciences, Shandong University of Traditional Chinese Medicine, Jinan, China; ^2^ Phase I Clinical Trial Ward, The Fifth Medical Center of Chinese the PLA General Hospital, Beijing, China; ^3^ Treatment and Research Center for Infectious Diseases, The Fifth Medical Center of PLA General Hospital, National Clinical Research Center for Infectious Diseases, Beijing, China; ^4^ Department of Gastroenterology of Chinese PLA General Hospital, Beijing, China; ^5^ Department of Infectious Diseases, The Fifth Medical Center of Chinese PLA General Hospital, National Clinical Research Center for Infectious Diseases, Beijing, China; ^6^ Respiratory Department No. 960 Hospital, The PLA, Jinan, China

**Keywords:** novel coronavirus pneumonia, convalescent plasma therapy, COVID-19 nucleic acid turned negative, negative conversion rate, retrospective analysis, multifactor analysis

## Abstract

**Objective:**

This study investigates the association between convalescent plasma therapy and the negative conversion rate in patients with persistent COVID-19 test positivity.

**Method:**

A retrospective analysis was conducted on patients with severe or mild to moderate COVID-19 whose viral nucleic acid tests remained positive for over 30 days. Patients were categorized into two groups: those who administered convalescent plasma therapy and those who were not. Data collected included information on therapy strategies used (convalescent plasma, corticosteroids, interferons, etc.), patients’ demographic characteristics, comorbidities, therapeutic medications, and nucleic acid testing results. Patients in the convalescent plasma therapy group were matched 1:2 ratio with those in the non-convalescent plasma therapy group. Cumulative negative conversion rates on the fifth, tenth, and fifteenth days post-therapy initiation were analyzed as dependent variables. Independent variables included therapy strategies, demographic characteristics, comorbidities, and therapeutic medication usage. Univariate analysis was conducted, and factors with a *p*-value (*P*) less than 0.2 were included in a paired Cox proportional hazards model.

**Results:**

There was no statistically significant difference in the cumulative negative conversion rate between the convalescent plasma therapy group and the non-convalescent plasma therapy group on the fifth, tenth, and fifteenth days. Specifically, on day the fifth, the negative conversion rate was 41.46% in the convalescent plasma therapy group compared to 34.15% in the non-convalescent plasma therapy group (HR: 1.72, 95% CI: 0.82–3.61, *P* = 0.15). On the tenth day, it was 63.41% in the convalescent plasma therapy group and 63.41% in the non-convalescent plasma therapy group (HR: 1.25, 95% CI: 0.69∼2.26, *P* = 0.46). On the fifteenth day, the negative conversion rate was 85.37% in the convalescent plasma therapy group and 75.61% in the non-convalescent plasma therapy group (HR: 1.19, 95% CI: 0.71–1.97, *P* = 0.51).

**Conclusion:**

Our finding does not support the hypothesis that convalescent plasma therapy could accelerate the time to negative conversion in patients who consistently test positive for COVID-19.

## 1 Introduction

Coronavirus disease 2019 (COVID-19) is a severe atypical respiratory infection first reported in Wuhan, China, in December 2019. COVID-19 is an infectious disease caused by the coronavirus COVID-19, which can cause a variety of respiratory diseases, colds, fever, and other symptoms, with a high infection rate, rapid mutation rate, low blood oxygen in the human body (Ar Gouilh et al., 2018; Gattinoni et al., 2021; Ochani et al., 2021; Rahman et al., 2021). The disease has rapidly spread around the world, causing a great impact on the global economy and serious damage to human health, resulting in millions of confirmed cases and hundreds of thousands of deaths (Gavriatopoulou et al., 2021).

In the face of the sudden onset of COVID-19, clinicians have adopted a variety of aggressive therapy strategies, including the use of Chinese medicine, thymosin, interferon, and convalescent plasma therapy (Dai et al., 2020; Chen et al., 2021; Huang et al., 2021; Smith et al., 2022; Bellet et al., 2023). Convalescent plasma therapy is an important means of therapy that has many benefits for patients, can effectively shorten the discharge time and improve the symptoms of patients (Janiaud et al., 2021). In the previous outbreak of severe acute respiratory syndrome (SARS) caused by SARS-CoV-1 coronavirus, studies have shown that convalescent plasma therapy can effectively shorten the course of disease, accelerate recovery, and reduce mortality (Zhou X. et al., 2003; Berger et al., 2004; Cheng et al., 2005).

At present, there have been several clinical studies on convalescent plasma therapy, and there have been some controversies. For example, some studies have found that there is no significant correlation between convalescent plasma therapy and the mortality of COVID-19 patients, and the survival and discharge rates of patients in the convalescent plasma therapy group and the non-convalescent plasma therapy group are similar (RECOVERY Collaborative Group, 2021). However, one study found that convalescent plasma therapy can effectively reduce the mortality of patients (Liu Z, 2020), and another study has shown that convalescent plasma therapy can improve the rate of negative conversion in COVID-19 patients over 60 days (Duan et al., 2022; Pan et al., 2022). Therefore, we conducted this study to investigate the relationship between convalescent plasma therapy and the viral clearance rate in patients with prolonged COVID-19 positivity.

## 2 Materials and methods

### 2.1 Research overview

This study included all patients with severe or mild to moderate COVID-19 diagnosed in 2020 at Huo Shenshan Hospital and Taikang Tongji Hospital in Wuhan, Hubei Province, China. It has been reviewed and approved by the Medical Ethics Committee of the Fifth Medical Center of the PLA General Hospital (approval number: 2020075D).

### 2.2 Inclusion criteria

(1) Age >18 years old, gender unlimited; (2) All patients included in the study met the diagnostic criteria for COVID-19 (Lin and Li, 2020); (3) Severe or mild to moderate cases, the manifestations of fever and respiratory symptoms, imaging findings of pneumonia; (4) Nucleic acid positive duration more than 30 days; (5) Nucleic acid testing was performed every 1–3 days after 30 days in all patients to determine whether the nucleic acid turned negative; (6) COVID-19 detection is to extract nucleic acid from specimens using an automatic nucleic acid extraction instrument (KingFisher Flex, Thermo Company), collect purified nucleic acid for real-time fluorescent RT-PCR detection, and the period threshold (CT) value of 40 or greater is considered negative; (7) All patients had blood routine and biochemical examination records for 30 ± 3 days.

### 2.3 Exclusion criteria

(1) Patients with malignant tumors and malignant blood diseases; (2) Acquired immune deficiency syndrome (AIDS) patients; (3) Patients with liver failure or renal failure; (4) Patients with incomplete clinical information (see “Section 2.5”) (Lin and Li, 2020); (5) Other diseases that researchers believe may affect this study, such as lupus erythematosus.

### 2.4 Standard for negative conversion

Refer to “Diagnosis and Therapy Protocol for COVID-19 (Trial Fifth Revised Version)”, and negative nucleic acid tests of respiratory pathogens were performed two consecutive times with a time interval of 24 h or more.

### 2.5 Clinical Information collection

The following data were obtained from clinical records: (1) demographic data (gender, age); (2) concomitant diseases (such as diabetes, hypertension, cardiovascular and cerebrovascular diseases, respiratory diseases, chronic liver diseases, hematopoietic diseases, etc.); (3) The status of therapeutic drugs; (4) nucleic acid test negative time (see negative criteria); (5) 30 ± 3 days blood routine and blood biochemical test results; (6) Other therapy strategies.

### 2.6 Case matching method

The convalescent plasma therapy group and the non-convalescent plasma therapy group were matched in ratio of 1:2 by therapy time (date of convalescent plasma therapy minus date of onset). The matching method was as follows: The convalescent plasma therapy group was sorted according to the time interval between onset and convalescent plasma therapy. Patients in the convalescent plasma therapy group with the longest time interval between the therapy date and the onset date were first selected, and the patients with a negative conversion time longer than this interval were chosen from the non-convalescent plasma therapy group, and twice as many patients from the non-convalescent plasma therapy group over this were randomly selected for matching. The remaining patients entered the matching process with the second patient in the convalescent plasma therapy group, and so on. Until the last patient in the convalescent plasma therapy group was matched.

### 2.7 Statistical analysis

They were divided into two groups: the convalescent plasma therapy group, which received convalescent plasma therapy, and the non-convalescent plasma therapy group, which did not receive convalescent plasma therapy. The baseline conditions of the two groups were analyzed. mean ± SD was used to represent measurement data when they met normality, and a T-test was used for comparison between groups. When the measurement data did not conform to normality, the median (IQR) was used, and the Wilcoxon rank sum test was applied for inter-group comparison. The patients were grouped according to whether they turned negative on the fifth day, tenth day, or fifteenth day after receiving convalescent plasma therapy, and univariate analysis was conducted to explore the relationship between the respective variables and the dependent variables under univariate conditions and to provide a basis for the selection of independent variables. The statistical analysis method is the same as above. With whether or not the nucleic acid turned negative on the fifth day, tenth day, and fifteenth day as the dependent variable, variables with *P* < 0.20 in the above univariate analysis were selected as independent variables. The Cox proportional risk model was used to analyze the relationship between convalescent plasma therapy and nucleic acid turning negative after adjusting for the influence of other independent variables. All the above statistical analysis processes were completed based on SAS 9.4, and both were adopted by a two-sided test (α = 0.05).

## 3 Result

**FIGURE 1:**
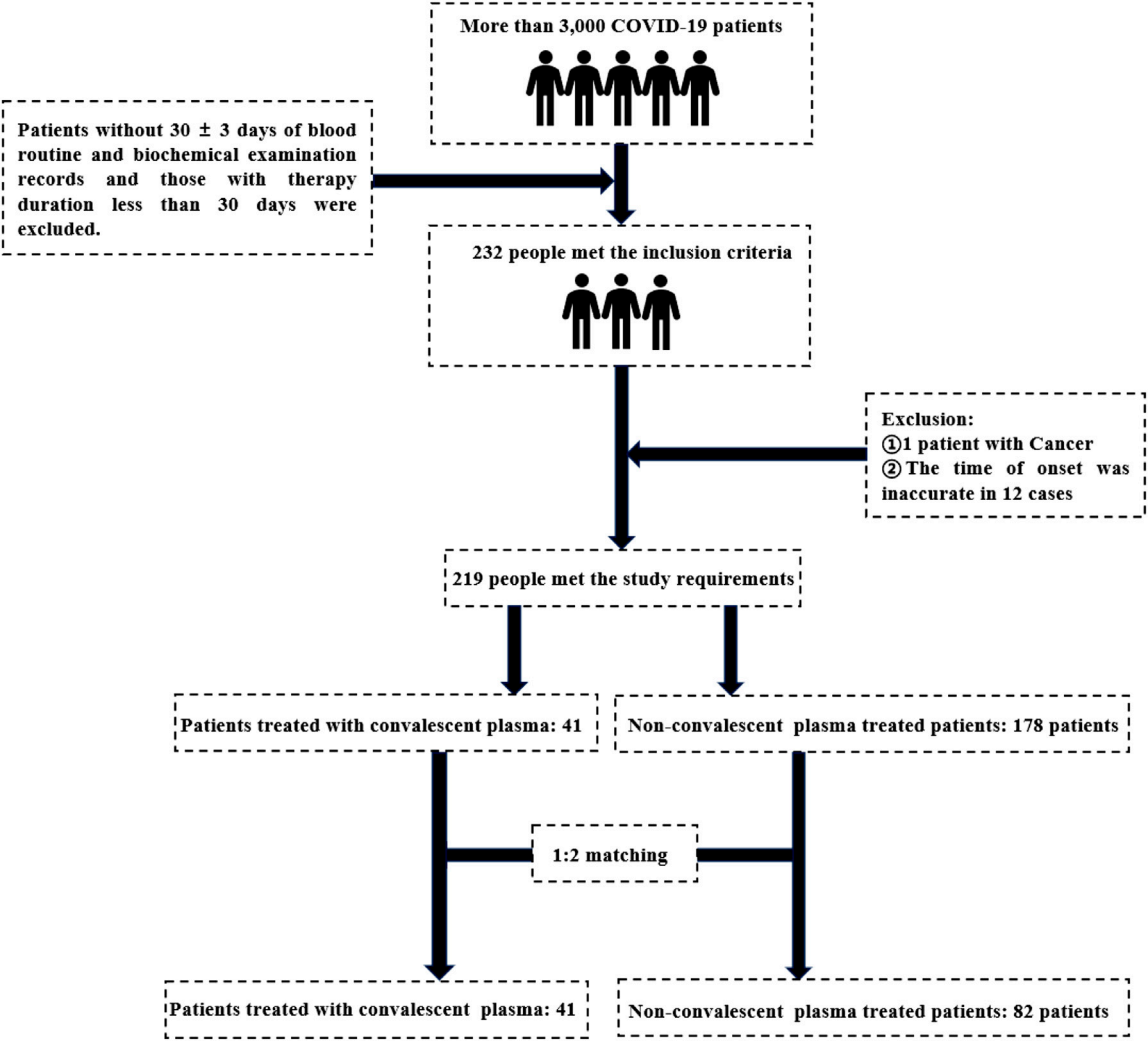
The process of case matching.

The initial sample size was 3,000 patients. After excluding the patients without 30 ± 3 days of routine blood and biochemical examination records and the therapy time was less than 30 days, a total of 232 patients meeting the inclusion criteria were enrolled in the study. Excluding tumor patients and 12 patients with inaccurate specific onset time, 219 patients met the requirements of this study, of which 41 patients received convalescent plasma therapy. After 1:2 matching, 123 patients were finally included, including 41 patients in the convalescent plasma therapy group and 82 patients in the non-convalescent plasma therapy group (see Figure 1).

### 3.1 Baseline data of patients in the convalescent plasma therapy group and non-convalescent plasma therapy group

The results showed that there were significant differences in platelet (PLT, *P* = 0.01), diabetes (*P* = 0.003), cardiovascular and cerebrovascular diseases (*P* = 0.04), interferon use (INF, *P* = 0.009), and glucocorticoid therapy (*P* = 0.03) between the two groups, suggesting that the baseline of the two groups was unbalanced (see Table 1).

**TABLE 1:** Baseline data of patients treated with and without therapeutic convalescent plasma (*N* = 123).

Trail	Convalescent plasma therapy group (N = 41)	Non-convalescent plasma therapy group (N = 82)	*P* value
Age	57 (51, 69)	62 (51, 68)	0.18
Sex (n, %)			0.42
Male	33 (40.24%)	22 (53.66%)	
Female	49 (59.76%)	19 (46.34%)	
Severe or mild to moderate			0.99
Severe	16 (26.23%)	45 (73.77%)	
Mild to moderate	16 (26.23%)	45 (73.77%)	
Fever (n, %)	64 (78.50%)	28 (68.29%)	0.24
Clearance time (n, %)	55.6 (48.0, 63.0)	57.0 (47.0, 60.0)	0.68
Blood routine examination			
WBC (10^9^/L)	5.5 (4.5, 7.0)	5.7 (4.4, 7.1)	0.95
RBC (10^12^/L)	3.8 ± 0.6	3.8 ± 0.6	0.76
HGB (g/L)	116.5 ± 17.0	118.1 ± 20.1	0.65
PLT (10^9^/L)	228.6 ± 68.3	196.8 ± 62.5	0.01
NE (10^9^/L)	3.3 (2.6, 4.0)	3.4 (2.5, 4.6)	0.81
LYM (10^9^/L)	1.5 (1.1, 1.9)	1.5 (1.1, 2.0)	0.81
MON (10^9^/L)	0.5 (0.3, 0.6)	0.4 (0.3, 0.6)	0.86
Blood biochemical examination			
ALT (IU/L)	18.4 (11.9, 34.2)	20.9 (13.4, 40.4)	0.42
AST (IU/L)	18.2 (14.3, 23.0)	18 (14.4, 24.8)	0.85
TP (g/L)	65.4 (62.2, 71.0)	65.9 (61.3, 68.7)	0.66
ALB (g/L)	37.7 (35.0, 40.3)	38.0 (36.2, 39.2)	0.99
GLO (g/L)	27.3 (25.2, 30.5)	28 (24.9, 30.4)	0.54
A/G	1.4 ± 0.2	1.4 ± 0.3	0.29
EOS (10^9^/L)	0.2 (0.02, 0.85)	0.2 (0.01, 0.6)	0.91
BASO (10^9^/L)	0.03 (0.01, 0.12)	0.03 (0.01, 0.05)	0.97
LDH (IU/L)	186.2 (107.0, 325.9)	218.0 (118.9, 637.9)	0.89
CK (IU/L)	61.6 (11.8, 316.8)	57.5 (12.7, 133.7)	0.95
BUN (mg/dL)	4.8 (1.7, 12.7)	4.9 (2.0, 12.3)	0.87
TBIL (umol/L)	9.7 (8.2, 12.9)	9.7 (8.0, 13.5)	0.82
DBIL (umol/L)	2.9 (2.3, 3.4)	3.5 (2.6, 4.9)	0.07
BUN (mmol/L)	4.2 (3.5, 5.2)	4.3 (3.9, 5.5)	0.44
CR (umol/L)	58.6 (51.2, 72.1)	62.0 (52.5, 74.3)	0.48
ALP (IU/L)	68.3 (57.9, 82.7)	70.3 (58.8, 81.6)	0.51
GGT (IU/L)	26.5 (17.1, 49.8)	26.1 (19.5, 49.8)	0.57
Concomitant disease (n, %)			
Hypertension	27 (32.93%)	16 (39.02%)	0.51
Diabetic	6 (7.41%)	11 (26.83%)	0.003
Cardiovascular disease	7 (8.54%)	9 (21.95%)	0.04
Therapeutic strategy (n, %)			
Thymosin	41 (50.00%)	24 (58.54%)	0.37
INF	3 (3.75%)	7 (8.40%)	0.33
Glucocorticoid	13 (15.85%)	13 (33.33%)	0.03

WBC, white blood cell count; RBC, red blood cell count; HGB, hemoglobin; PLT, platelet count; NE, neutrophil count; LYM, lymphocyte count; MON, monocyte count; ALT, alanine aminotransferase; AST, aspartate aminotransferase; TP, total protein; ALB, albumin; GLO, globulin; A/G, Albumin/Globulin Ratio; EOS, eosinophil count; BASO, basophil count; LDH, lactate dehydrogenase; CK, creatine kinase; BUN, blood urea nitrogen; TBIL, total bilirubin; DBIL, direct bilirubin; CR, creatinine; ALP, alkaline phosphatase; GGT, Gamma-Glutamyl Transferase.

### 3.2 Univariate analysis related to negative nucleic acid transformation on the fifth day, tenth day, and fifteenth day

The results showed that when the fifth-day cumulative conversion rate was used as the dependent variable, the independent variables with *P* < 0.20 were neutrophil (NE, *P* = 0.17), Glutamic-pyruvic transaminase (ALT, *P* = 0.19), Glutamic oxalacetic transaminase (AST, *P* = 0.07) and cardiovascular and cerebrovascular diseases (*P* = 0.11). When the tenth day cumulative conversion rate was used as the dependent variable, the independent variables with *P* < 0.20 were sex (*P* = 0.14), hemoglobin (HGB, *P* = 0.19), Glutamic-pyruvic transaminase (ALT, *P* = 0.11) and the ratio of albumin to globulin (A/G, *P* = 0.17). On the fifteenth day, when cumulative conversion rate was a factor variable, the self-variability of *P* < 0.20 was HGB (*P* = 0.18) and direct bilirubin (DBIL, *P* = 0.17) (see Table 2).

**TABLE 2:** Univariate analysis of negative transformation on the fifth day, tenth day, and fifteenth day.

Fifth day				Tenth day			Fifteenth day		
Trait	Non-negative conversion (N = 82)	Negative conversion (N = 41)	*P* value	Non-negative conversion (N = 82)	Negative conversion (N = 41)	*P* value	Non-negative conversion (N = 82)	Negative conversion (N = 41)	*P* value
Age	63 (51, 69)	63 (55, 69)	0.60	63 (49, 69)	62 (54, 69)	0.75	66.5 (52, 70)	61 (51, 69)	0.54
Sex (n, %)			0.42			0.14			0.54
Male	37 (47.44%)	18 (40.00%)		24 (53.33%)	31 (39.74%)		13 (50.00%)	42 (43.30%)	
Female	41 (52.56%)	27 (60.00%)		21 (46.67%)	47 (60.26%)		13 (50.00%)	55 (56.70%)	
Fever (n, %)	64 (78.50%)	28 (68.29%)	0.24	31 (68.89%)	61 (78.21%)	0.25	18 (69.23%)	74 (76.29%)	0.46
Blood routine examination									
WBC (109/L)	5.5 (4.5, 7)	5.7 (4.4, 7.1)	0.95	5.6 (4.0, 5.7)	5.6 (4.5, 7.1)	0.89	5.7 (4.5, 7.2)	5.6 (4.5, 7.0)	0.76
RBC (1,012/L)	3.8 ± 0.6	3.8 ± 0.5	0.80	3.9 ± 0.6	3.8 ± 0.6	0.48	3.9 ± 0.6	3.8 ± 0.6	0.48
HGB (g/L)	117.2 ± 19.1	116.7 ± 16.3	0.89	119.8 ± 17.4	115.4 ± 18.3	0.19	121.3 ± 18.4	115.9 ± 17.8	0.18
PLT (109/L)	217.8 ± 68.7	218.3 ± 67.1	0.97	209.3 ± 74.3	223.0 ± 63.8	0.28	216.0 ± 77.2	218.6 ± 65.5	0.86
NE (109/L)	3.3 (2.4, 4.2)	3.4 (2.9, 4.5)	0.17	3.2 (2.2, 4.0)	3.4 (2.6, 4.5)	0.24	3.2 (2.4, 4.2)	3.4 (2.6, 4.3)	0.67
LYM (109/L)	1.6 (1.1, 1.9)	1.5 (1.1, 2.3)	0.90	1.7 (1.3, 1.9)	1.4 (1.1, 2.0)	0.28	1.6 (1.3, 1.9)	1.5 (1.1, 2.0)	0.66
MON (109/L)	0.4 (0.3, 0.6)	0.5 (0.4, 0.6)	0.33	0.4 (0.3, 0.5)	0.5 (0.4, 0.6)	0.26	0.4 (0.3, 0.6)	0.5 (0.4, 0.6)	0.44
Blood biochemical examination									
ALT (IU/L)	18.4 (12.1, 37.0)	15.9 (11.7, 30.7)	0.19	27.3 (13.4, 8.0)	16.7 (11.4, 32.4)	0.11	29.2 (12.3, 38.6)	17.6 (12.3, 38.6)	0.30
AST (IU/L)	18.6 (15.2, 25.0)	16.7 (13.5, 22.4)	0.07	18.7 (15.2, 25.0)	17.5 (1,400, 23.5)	0.28	20.8 (14.5, 26.4)	18.0 (14.3, 3.2)	0.40
TP (109/L)	66.0 ± 6.9	65.7 ± 5.1	0.79	65.7 ± 5.9	66.0 ± 6.5	0.80	65.7 ± 5.9	66.0 ± 6.4	0.88
ALB (109/L)	37.6 ± 4.4	37.6 ± 3.1	0.97	37.9 ± 3.9	37.4 ± 4.0	0.46	37.3 ± 4.0	37.6 ± 4.0	0.64
GLO (109/L)	28.0 (25.2, 30.5)	27.1 (24.9, 30.4)	0.49	27.3 (25.2, 30.1)	27.5 (25.0, 31.6)	0.54	27.6 (25.5, 29.2)	27.5 (24.9, 30.5)	0.86
A/G	1.4 ± 0.3	1.4 ± 0.3	0.51	1.4 (1.3, 1.6)	1.4 (1.2, 1.5)	0.17	1.4 ± 0.3	13.7 ± 0.2	0.98
EOS (109/L)	0.2 (0.04, 0.4)	0.2 (0.01, 0.6)	0.58	0.2 (0.04, 0.9)	0.2 (0.01, 0.6)	0.64	0.2 (0.04, 0.9)	0.2 (0.01, 0.6)	0.69
BASO (109/L)	0.03 (0.01, 0.1)	0.03 (0.01, 0.05)	0.68	0.03 (0.01, 0.1)	0.03 (0.01, 0.05)	0.69	0.03 (0.01, 0.1)	0.03 (0.01, 0.05)	0.72
LDH (IU/L)	183.3 (120.1, 325.0)	218.6 (118.9, 493.8)	0.78	186.7 (120.1, 325.0)	218.6 (118.9, 493.8)	0.77	186.7 (120.1, 325.0)	217.9 (118.9, 637.9)	0.97
CK (IU/L)	63.2 (19.1, 316.8)	57.3 (12.7, 133.7)	0.91	61.5 (11.8, 316.8)	57.3 (12.7, 316.8)	0.89	61.5 (11.8, 316.8)	56.7 (12.7, 133.7)	0.87
BUN (mg/dL)	4.5 (2.63, 5.85)	4.7 (4.1, 8.5)	0.88	4.5 (2.6, 5.9)	4.7 (3.4, 8.5)	0.65	4.5 (2.6, 10.5)	4.8 (3.4, 8.5)	0.88
TBIL (umol/L)	9.8 (8.1, 12.9)	9.4 (8.0, 13.5)	0.99	9.6 (8.1, 12.6)	9.7 (8.0, 13.5)	0.99	10.6 (8.6, 12.6)	9.5 (8.0, 13.4)	0.55
DBIL (umol/L)	3.2 (2.4, 4.2)	3 (2.5, 4.5)	0.86	3.3 (2.4, 4.2)	3.0 (2.5, 4.3)	0.43	3.6 (2.5, 4.6)	3.0 (2.3, 3.9)	0.17
BUN (umol/L)	4.4 (3.8, 5.5)	4.06 (3.5, 5.1)	0.22	4.3 (3.9, 5.5)	4.3 (3.7, 5.2)	0.62	4.6 (3.9, 5.9)	4.3 (3.7, 5.2)	0.25
CR (umol/L)	60.4 (51.2, 73.3)	58.7 (52.5, 70.1)	0.51	59.4 (52.1, 76.7)	60.4 (51.3, 72.5)	0.82	59.2 (51.2, 81.3)	60.3 (51.4, 72.5)	0.58
ALP (IU/L)	70.0 (57.9, 91.9)	68.6 (58.8, 79.6)	0.53	69.6 (54.4, 85.1)	68.7 (58.0, 81.6)	0.87	70.6 (54.4, 81.9)	68.8 (58.0, 82.7)	0.85
GGT (IU/L)	28.0 (19, 47.4)	24.9 (17.8, 53.8)	0.83	27.4 (16.2, 44.0)	25.6 (18.4, 53.7)	0.50	28.1 (16.2, 44.0)	25.6 (18.4, 53.7)	0.58
Concomitant disease (n, %)									
Hypertension	28 (35.90%)	15 (33.33%)	0.77	13 (28.89%)	30 (38.45%)	0.28	9 (34.62%)	34 (35.05%)	0.97
Diabetes	11 (14.10%)	6 (16.64%)	0.94	5 (11.11%)	12 (15.58%)	0.49	3 (11.54%)	14 (14.58%)	0.99
Cardiovascular Disease	13 (16.67%)	3 (6.67%)	0.11	7 (15.56%)	9 (11.54%)	0.52	4 (15.38%)	12 (12.37%)	0.74
Therapeutic strategy (n, %)									
Thymosin	41 (51.28%)	25 (55.56%)	0.65	25 (55.56%)	40 (51.28%)	0.65	16 (61.54%)	49 (505.2%)	0.32
INF	16 (20.51%)	7 (15.56%)	0.50	9 (20.00%)	14 (17.95%)	0.78	5 (19.23%)	18 (18.54%)	0.99
Glucocorticoid	16 (21.05%)	10 (22.22%)	0.88	16 (21.05%)	10 (22.22%)	0.88	6 (23.08%)	20 (21.05%)	0.82
Convalescent plasma	28 (62.22%)	17 (37.78%)	0.43	52 (66.67%)	26 (33.33%)	0.99	62 (63.92%)	35 (36.08%)	0.21

### 3.3 Multi-factor analysis related to negative nucleic acid transformation on the fifth day, tenth day, and fifteenth day

The Cox proportional risk model was applied to stratify the matched groups, with cumulative conversion rate on the fifth day as the dependent variable, and indexes with *P* < 0.20 in Table 2, including NE, ALT, AST, cardiovascular and cerebrovascular diseases, as the adjusting variables. There was no statistical significance between the convalescent plasma therapy group and the non-convalescent plasma therapy group (HR: 1.72, 95% CI: 0.82–3.61, *P* = 0.15). With the cumulative conversion rate on the tenth day as the dependent variable and the indexes with *P* < 0.20 in the results in Table 2, including HGB, ALT, A/G, and gender as the adjusting variables, there was no significant statistical significance between the convalescent plasma therapy group and the non-convalescent plasma therapy group (HR: 1.25, 95% CI: 0.69–2.26, *P* = 0.46); With the cumulative conversion rate on the fifteenth day as the dependent variable and the indexes with *P* < 0.20 in the results of Table 2, including HGB and DBIL as the adjusted variables, there was no significant statistical significance between the convalescent plasma therapy group and the non-convalescent plasma therapy group (HR: 1.19, 95% CI: 0.72–1.97, *P* = 0.51) (see Table 3).

**TABLE 3:** Multivariate Cox analysis of influencing factors related to the fifth day, tenth day, and fifteenth day cumulative conversion rate.

	Trait	HR	95% CI	*P*
Fifth day	NE (10^9^/L)	0.99	0.83–1.17	0.89
	ALT (IU/L)	1.00	0.98–1.03	0.76
	AST (IU/L)	1.00	0.93–1.07	0.94
	Convalescent plasma	1.72	0.82–3.61	0.15
	Cardiovascular Disease	0.31	0.08–1.22	0.09
Tenth day	HGB (g/L)	1.00	0.98–1.02	0.95
	ALT (IU/L)	0.99	0.98–1.01	0.28
	A/G	0.45	0.10–2.10	0.31
	SEX	0.90	0.46–1.77	0.76
	Convalescent plasma	1.25	0.69–2.26	0.46
Fifteenth day	HGB (g/L)	0.99	0.98–1.01	0.43
	DBIL (umol/L)	1.02	0.83–1.24	0.88
	Convalescent plasma	1.19	0.72–1.97	0.51

### 3.4 Comparison of Kaplan-Meier curve of negative conversion rate between the two groups

**FIGURE 2:**
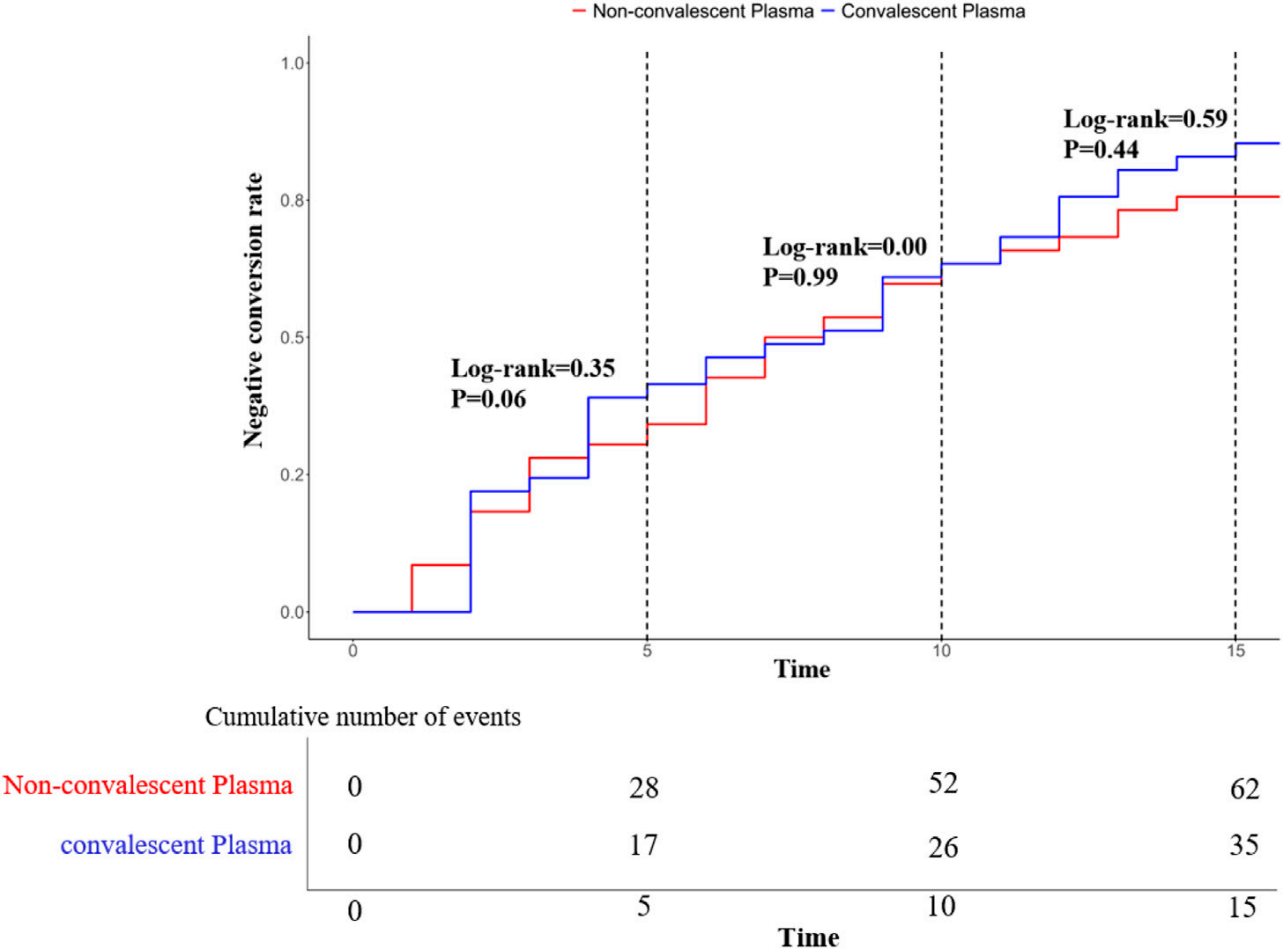
Kaplan-Meier curve of negative conversion rate in two groups.

The Kaplan-Meier curve of negative conversion rate between the convalescent plasma therapy group and the non-convalescent plasma therapy group showed that *log-rank* = 0.35, *P* = 0.56 cumulative conversion rate on the fifth day was taken as the dependent variable. When the cumulative conversion rate on the tenth day was taken as the dependent variable, *log-rank* = 0.00, *P* = 0.99; When the cumulative conversion rate on the fifteenth day was taken as the dependent variable, *log-rank* = 0.59, *P* = 0.44. These results showed that there was no statistical significance in Kaplan-Meier negative conversion rate curves between the convalescent plasma therapy group and the non-convalescent plasma therapy group on day 5, day 10, and day 15 (see Figure 2).

## 4 Discussion

The COVID-19 pandemic represents one of the most significant public crises in recent years. Determining the efficacy of convalescent plasma therapy in converting consistently COVID-19 positive patients to negative status is of paramount importance. To address this issue, we conducted a retrospective analysis and performed a cohort study on two groups of patients: those who received convalescent plasma therapy and those who did not receive convalescent plasma therapy. The convalescent plasma therapy group and the non-convalescent plasma therapy group were matched in 1:2 by therapy time (date of convalescent plasma therapy minus date of onset). The matching method was as follows: The convalescent plasma therapy group was sorted according to the time interval between onset and convalescent plasma therapy. Patients in the convalescent plasma therapy group with the longest time interval between the therapy date and the onset date were first selected, and the patients with a negative conversion time longer than this interval were chosen from the non-convalescent plasma therapy group, and twice as many patients from the non-convalescent plasma therapy group over this were randomly selected for matching. The remaining patients entered the matching process with the second patient in the convalescent plasma therapy group, and so on. Until the last patient in the convalescent plasma therapy group was matched. Initially, we performed univariate analysis on all independent variables. Factors with a *P* < 0.20 from the univariate analysis were then included in the Cox proportional hazards model for multivariate analysis. The results showed that after adjusting for other factors (NE, ALT, AST, cardiovascular and cerebrovascular diseases), we still did not find a statistical difference between therapy with convalescent plasma and therapy without convalescent plasma.

Our study does not support the association between convalescent plasma therapy and acceleration of SARS-CoV-2 conversion among COVID-19 patients with persistent positive RT-PCR tests. During the 2002 outbreak of Severe Acute Respiratory Syndrome (SARS), convalescent plasma therapy was shown to significantly facilitate the conversion of patients who continued to test positive for the SARS virus to negative (Berger et al., 2004). Therefore, during the current COVID-19 pandemic, many clinicians have also employed convalescent plasma therapy to expedite viral clearance in patients. Recent years have seen numerous randomized clinical trials indicating that convalescent plasma therapy shows no significant association with clinical outcomes (Discharge, death, etc.) in patients with persistent positive COVID-19 test results (Agarwal et al., 2020b; Li et al., 2020; AlQahtani et al., 2021; Balcells et al., 2021; O’Donnell et al., 2021; Simonovich et al., 2021; Iannizzi et al., 2023). This aligns with the findings in the WHO COVID-19 Therapy Guidelines regarding convalescent plasma therapy (Agarwal et al., 2020a). However, these studies have not specifically investigated whether convalescent plasma therapy is related to the transition to negative test results in these patients. Our study further supplemented the evidence, demonstrating that convalescent plasma therapy is ineffective in accelerating viral clearance in patients who remain persistently positive for COVID-19. This finding is significant for the therapy of COVID-19 patients, as it suggests that convalescent plasma may not effectively shorten the recovery period or promote patient recuperation. We recommend incorporating these findings into current therapy protocols, which may lead clinicians to use convalescent plasma prudently when treating patients with persistent COVID-19 test positivity, not only to avoid the risk of donation from donors, but also to reduce the risk of ineffective therapy for patients.

Previous research on convalescent plasma therapy for COVID-19 has highlighted its potential benefits and limitations, warranting further investigation into its clinical efficacy and safety. Some studies have shown that administering convalescent plasma from recovered patients to severely ill individuals can rapidly provide immunoglobulins, allowing for timely therapy and potentially becoming an important therapeutic measure, especially for those with compromised immunity (Ouyang et al., 2020; Senefeld et al., 2023). Additionally, early-stage administration of convalescent plasma therapy has been suggested to offer clear clinical benefits due to the rapid viral replication and high viral load in patients (Sun et al., 2020). Our data included both severe patients with COVID-19 and those with early-stage COVID-19 infection, but in neither case did convalescent plasma therapy demonstrate a positive effect on clinical outcomes. Moreover, convalescent plasma therapy remains an “experimental therapy” in clinical practice (Zeller et al., 2015). Its composition, which includes various components, can lead to serious medical complications such as allergic reactions in recipients (Shu et al., 2021; Wood et al., 2021; Sullivan et al., 2022; Senefeld et al., 2023). Compared to small molecule drugs, convalescent plasma therapy is less safe and lacks sufficient clinical research (Chai et al., 2020). Therefore, extensive prospective clinical trials are necessary to further explore its efficacy and safety.

The advantage of this study is that we used data from Wuhan, where all COVID-19 patients were systematically and centrally isolated and treated, and comprehensive patient data were obtained. Each patient was continuously observed until they tested negative for nucleic acid and discharged, providing a complete and continuous dataset from admission to discharge. However, this study also has several limitations: 1) Although the initial sample size was large, with over 3,000 cases, only 219 cases met the study criteria, and after matching, only 123 cases were included, making it a small retrospective cohort study; 2) Due to the inherent limitations of retrospective studies, the measured factors included in the analysis may not be complete, and some unknown factors may lead to bias in the results.

## 5 Conclusion

This study does not support that convalescent plasma therapy is associated with acceleration of negative conversion in COVID-19 patients with persistent positive RT-PCR tests.

## Data Availability

The original contributions presented in the study are included in the article/Supplementary Material, further inquiries can be directed to the corresponding authors.
